# Retinal and Preretinal Hemorrhages in a Patient Receiving Hyper-CVAD Chemotherapy for T-Cell Acute Lymphoblastic Leukemia

**DOI:** 10.1155/2018/9457549

**Published:** 2018-12-03

**Authors:** Krishi Peddada, Stephanie J. Weiss, Shaina Kumar, Deepika Malik

**Affiliations:** Department of Ophthalmology, Drexel University College of Medicine, Philadelphia, PA, USA

## Abstract

Hyperfractionated cyclophosphamide, vincristine, adriamycin, and dexamethasone (Hyper-CVAD) is an important chemotherapeutic regimen for acute lymphoblastic leukemia (ALL) and non-Hodgkin's lymphoma. We present a case of a 23-year-old male with T-cell ALL and visual acuity of 20/20 in the right eye and 20/25 in the left eye who developed significant changes in his vision after starting Hyper-CVAD therapy. The patient initially presented with cotton wool spots in the fundus shortly after starting the regimen. After going through the induction phase of chemotherapy, he had a sudden decline in his vision to light perception in the left eye. Posterior segment exam revealed retinal ischemia and multilayered hemorrhages in both eyes as well as a large preretinal hemorrhage obscuring the fovea in the left eye. Labs associated the appearance of these hemorrhages with a significant decrease in hemoglobin and a platelet count of 5 K/*μ*L. A Nd:YAG laser applied in the left eye at the posterior hyaloid face allowed blood to drain into the vitreous cavity and brought the patient's visual acuity back to baseline. Hyper-CVAD is an aggressive chemotherapy regimen that can cause severe thrombocytopenia secondary to myelosuppression. Frequent retinal evaluations and timely intervention is advisable in these cases as extensive intraretinal hemorrhages may cause irreversible damage.

## 1. Introduction

Acute lymphoblastic leukemia (ALL) significantly alters blood counts in the body, leaving vulnerable areas such as retinal microvasculature susceptible to damage [1]. These changes are known as secondary ocular manifestations of ALL (multilayered retinal hemorrhages, cotton-wool spots, Roth spots, and vascular occlusions) and they occur in 31.6-64.2% of patients [2–6]. The initiation of chemotherapy to treat ALL can potentially worsen ocular manifestations, as chemotherapeutic agents have been associated with side effects in many compartments of the eye [7]. Case reports demonstrate that cisplatin and etoposide are associated with retinal toxicities, as demonstrated by changes in electroretinogram and visually evoked potentials in children [8]. Vincristine causes damage to the optic nerve and ocular motor nerves [9]. Methotrexate has been implicated in central neurotoxicity [10]. The role of the ophthalmologist in management of ocular changes either from leukemia or chemotherapy remains poorly defined and without specific monitoring guidelines [6].

Intensive, multiagent chemotherapeutic regimens are now being widely used for aggressive leukemias [11]. Hyperfractionated cyclophosphamide, vincristine, adriamycin, and dexamethasone (part A) alternating with methotrexate and cytarabine (part B) (Hyper-CVAD) are one such regimen that is used in treating certain types of acute lymphoblastic leukemia and non-Hodgkin's lymphoma. This treatment is administered every two to three weeks for a total of four cycles [11] and can achieve remission in 80-93.8% of cases [11–13]. Systemic side effects of Hyper-CVAD (prolonged myelosuppression and resultant pancytopenia) are common and seen in up to 59% of patients in three months of treatment [14, 15]. Because ALL patients that are already predisposed to retinopathy are on Hyper-CVAD, it has been difficult to link ocular changes to Hyper-CVAD definitively. The case reports that have been successful at establishing a connection have focused on ocular changes manifested by Hyper-CVAD-induced myelosuppression, such as activation of opportunistic infections [16].

## 2. Case Report

**Figure 1:**
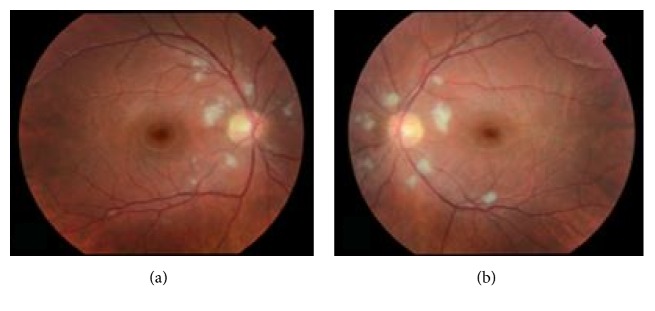
Multiple peripapillary cotton wool spots in both eyes at presentation.

We report the case of a 23-year-old male with T-cell ALL undergoing treatment with Hyper-CVAD that presented initially with blurry vision. Upon presentation in August 2016, the patient was 19 days status after treatment cycle 1B of his Hyper-CVAD therapy. His hemoglobin level was 10.5 mg/dL and his platelet count was 63 K/*μ*L on presentation in the eye clinic. On examination, the patient was found to have best corrected Snellen visual acuity of 20/20 in the right eye (OD) and 20/25 in the left eye (OS). Anterior segment examination of both eyes (OU) was unremarkable. Fundoscopic examination OU revealed multiple peripapillary cotton wool spots in both eyes (Figures 1(a) and 1(b)). There was no evidence of hemorrhage or leukemic infiltration. At this time, observation was recommended.

**Figure 2:**
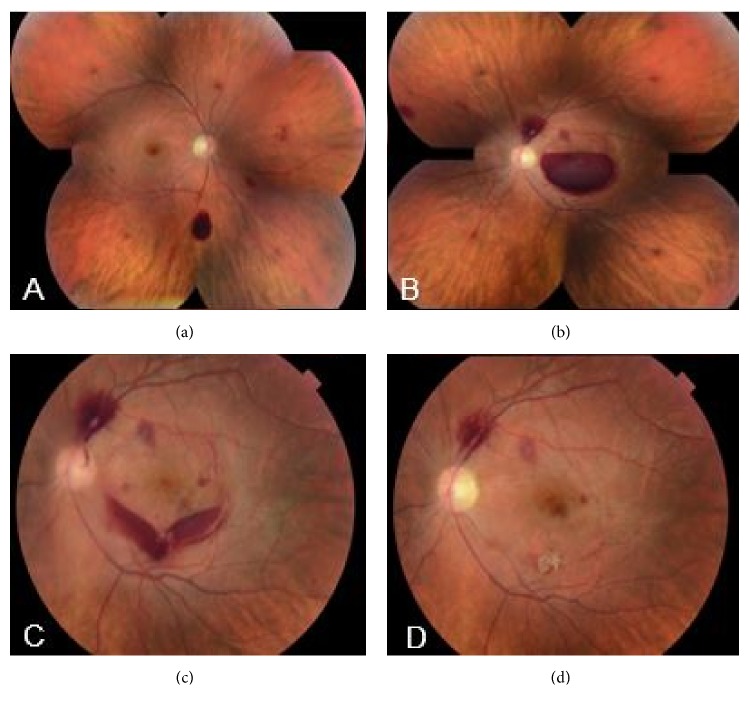
Following Hyper-CVAD chemotherapy, (a) multiple preretinal, intraretinal, and subretinal hemorrhages; (b) preretinal, intraretinal, and subretinal hemorrhages in the left eye; (c) after YAG laser treatment in the left eye, blood settling inferiorly in the vitreous cavity; (d) the retinal hemorrhages resolved after several months.

In mid-September 2016, 18 days after Hyper-CVAD treatment cycle 2B, the patient presented with decreased vision OS for one week. His hemoglobin level decreased to 7.4 gm/dL from 10.5 gm/dL prior to his most recent treatment cycle and his platelet count decreased to 5 K/*μ*L from 63 K/*μ*L. Despite clinical evidence of regression of the leukemia, he was found to have best corrected Snellen visual acuity of 20/20 OD and light perception OS. Anterior segment examination was within normal limits in both eyes. Fundoscopic examination revealed retinal hemorrhages extending from the peripapillary region into the midperipheral retina OU (Figures 2(a) and 2(b)), with a large premacular hemorrhage in the left eye. The premacular hemorrhage was a well-organized clot at the time. Observation was recommended. However, upon follow-up one week later, the examination revealed discrete layering of the premacular hemorrhage. At that time, a neodymium-doped yttrium aluminum garnet (Nd:YAG) laser was used to disrupt the posterior hyaloid face. As a result, the hemorrhage was free to diffuse into the vitreous cavity and settle inferiorly (Figure 2(c)). The patient's vision returned to baseline immediately after the procedure. During the entire course of treatment, the patient did not receive any platelet and/or blood transfusions. However, his hemoglobin level improved to 9.4 gm/dL and platelet count to 43 K/*μ*L two months later. The remaining retinal hemorrhages resolved over several months (Figure 2(d)) and the patient completed the remainder of his Hyper-CVAD therapy without further ocular complications. Of note, there were no further episodes of severe anemia or thrombocytopenia.

## 3. Discussion

The Hyper-CVAD regimen is well known to exacerbate the anemia and thrombocytopenia associated with ALL [17]. This regimen impairs the ability to mobilize peripheral blood progenitor cells and causes significant hematopoietic progenitor cell injury [15]. The high dose of steroids incorporated into the Hyper-CVAD regimen is also thought to further exacerbate the existing myelosuppression [16]. These complications occur more commonly during induction therapy [11]. Our patient presented initially with cotton wool spots secondary to ALL. He later developed retinal hemorrhages after a striking decrease in hemoglobin levels and platelet count induced by Hyper-CVAD treatment. This sequence of events suggests that Hyper-CVAD may act as an inciting factor by inducing further myelosuppression and may increase the risk of retinal complications in patients with ALL. This is further supported by the absence of further ocular complications two months later when hemoglobin levels increased to > 9 gm/dL and platelets increased to > 43 K/*μ*L.

Additional studies are needed to further define this novel association between Hyper-CVAD therapy and retinal complications. However, our findings suggest that close observation with serial ophthalmological examinations may be warranted in patients undergoing Hyper-CVAD treatment for ALL.
